# A Return-to-Work Intervention for Prematurely Retired Depression or Anxiety Disorder Patients

**DOI:** 10.3389/fpsyt.2021.662158

**Published:** 2021-06-02

**Authors:** Eva Behrens-Wittenberg, Felix Wedegaertner

**Affiliations:** Department of Psychiatry, Social Psychiatry and Psychotherapy, Hannover Medical School, Hannover, Germany

**Keywords:** absenteeism, disability, depression, anxiety, mental health, work ability, occupational disability, return to work

## Abstract

**Background:** Depression and anxiety disorders are the most common cause for premature retirement of people of middle age. These people are expelled from the workforce. The following social disintegration can have an additional detrimental effect on subjects' psychological well-being which further reduces the chance to re-enter the workforce. Depression and anxiety in general need not be regarded as irreversible causes of disability. Therefore, long-term disability should be avoidable in many cases. This two-arm prospective controlled study tests a novel approach for those who have become economically inactive due to their illness with the goal to improve psychological well-being and return to work. Forty-one subjects were followed-up on over a period of 12 months and compared to 41 control cases. ANOVA for repeated measures showed that experimental subjects' psychological well-being and work ability was much better after the intervention than in the control group. These findings show that an individually tailored return-to-work intervention can be a useful therapeutic tool even after retirement.

## Introduction

With 19% mental health issues, primarily depression and anxiety, have a high prevalence within the working population (1). They are associated with long sick leave periods (2–5). Affective disorders are the most common cause of absenteeism from work among all psychiatric illnesses (6).

One of the major consequences of severe mental disorders is the loss of one's ability to work. Those affected also lose of the various positive aspects associated with work (7). As sense of accomplishment and effectiveness at work rise, feelings of exhaustion and cynicism, which are typical aspects of depression, decline (8). Good work mobilizes, provides a daily routine and has a stabilizing effect (9). It enables participation in society and provides a sense of purpose and identity (10). Work also provides financial security, which protects against social decline (11). Accordingly, people with mental issues who are not in the workforce lack a major aspect of recovery, namely “good” work. Even those employees with mental issues that cannot show their full potential benefit from working or returning to work rather than not doing so (12–14).

In nations with statutory pension insurance it is in the general interest to have means of tertiary prevention available because of the associated long-term sick leave or retirement (15, 16). In the case of mental disorders the need for these is even more pressing as their occurrence earlier in the life-span increases the associated cumulative costs (17).

Unsurprisingly, there is a plethora of interventions available. Those for people with mental illness are heterogeneous and difficult to compare (18). Work-directed interventions aim to reduce the negative impact of the psychological disorder on the ability to work. Clinical interventions include pharmacological treatment, psychotherapy, or a combination of both and target the symptoms of the psychological disorder which, in turn, may improve work ability. Rehabilitation by specialized care givers aims at cognitive restructuring.

After disability retirement all rehabilitation efforts typically end. From a nosological and economical viewpoint this seems to be wrong. If only a small percentage of those who have retired returned to the work force, the costs of the intervention would be outweighed by the savings in pension payments quite quickly. Consequently, the authors designed an intervention to take place after premature retirement. The intervention is described in detail below. In short, it targets those suffering from depression or anxiety and who ended in long-term occupational disability after unsuccessful completion of rehabilitation interventions. It focuses on the individual fit between personal resources, appropriate treatment and work-related factors with the goal of mental health improvement and voluntary vocational reintegration to foster social participation as a major factor of recovery. The intervention was evaluated with regard to mental health, work ability and return to work.

## Methods

### Design

**Figure 1:**
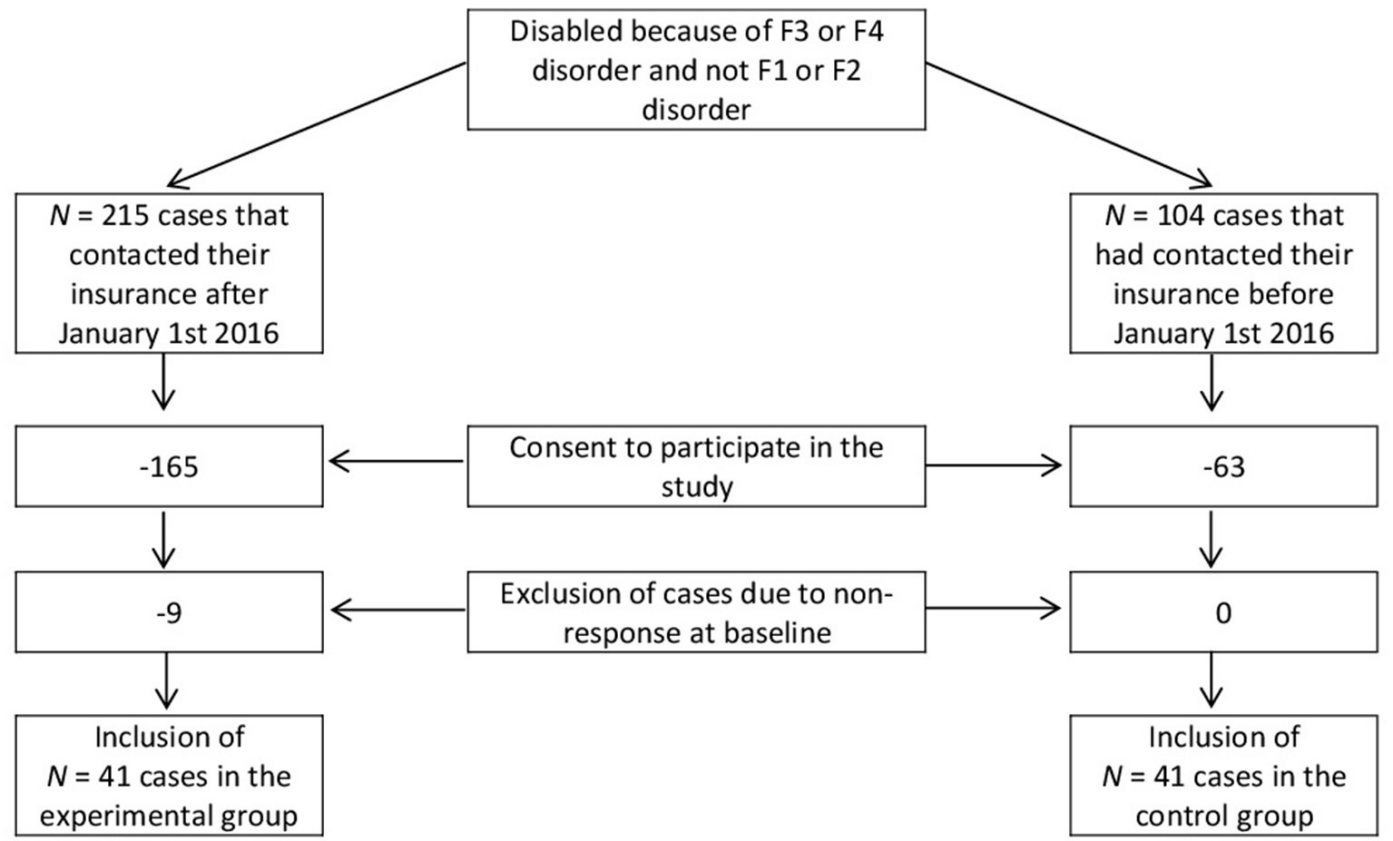
Sampling strategy.

Subjects of the experimental and control group were gathered from a pool of German-speaking life insurance clients regardless of their place of residence that received disability benefits due to an affective and/or neurotic disorder at any time between January 2012 and December 2019. All patients that contacted their insurance after January 1st 2016 and fulfilled inclusion criteria and had no exclusion criteria were offered the intervention. An equal number of controls were gathered from cases that had contacted their insurance before that date and followed parallel to the experimental cases. Cases were included until December 2019. Sample sizes at T0 for both groups counted *N* = 41. The flow chart (Figure 1) illustrates the sampling procedure.

After obtaining informed consent, subjects in the intervention and control group were observed for 12 months, while the intervention itself was done by a team of trained professionals. Data was collected using self-report questionnaires at the start of the observation (T0) after 6 months (T1) and after 12 months (T2). Included were all cases that had occupational disability due to an affective (ICD-10: F30-F39) and/or neurotic disorder (ICD-10: F40-F48). Inclusion criteria were selected as these mental illnesses are generally well-treatable and need not be regarded as irreversible causes of disability. Exclusion criteria were comorbid substance use disorders (ICD-10: F10-F19) and/or schizophrenic disorders (ICD-10: F20-F29). The morbidity was extracted from the subjects' doctor's notes, which were available.

### Procedure

The experimental group received the intervention which is described in detail below. The control group received no intervention and was observed. Subjects received 10 Euros with every questionnaire. Response rate was high in both groups and ranged from 93 to 98% for T1 and T2.

### Intervention

The intervention was conceptualized by the authors, payed for by the client's private life insurance and implemented by rehabilitation services (RS). The RS acted independently of the interests of any insurance company, employers or therapists. The resources necessary for the intervention were not provided by the subject's statutory health or pension insurance.

A psychologist and several case managers of the RS worked within the study and supported the subjects mainly via telephone and online. At least one home visit was provided to each subject. The intervention focused on the coordination and implementation of subjects' health care network, supportive coaching and return to work.

### Intervention Program

The main target of the intervention was an improved match between the subjects' abilities and requirements in daily tasks, treatment expectations, and reality. For this purpose a detailed assessment was conducted after which the subjects were supported by the RS' case manager and psychologist over a 1-year period, depending on task at hand and individual need. For every subject 80 h of intervention were available. The intervention was semi-standardized in so far as return-to-work was the main goal, but the single steps of the intervention were always oriented at the individual case. See Table 1 for an overview of the methods at hand.

**Table 1:** Steps of the intervention program of the rehabilitation services.

**Step**	**Focus**	**Actions**
1	Health care network	•Needs assessment •Preparation of information on treatment options •Investigation of discipline-specific and local treatment units (psychiatrists, psychotherapist, ergotherapy, etc.) as well as first contact, if desired •Arrangement of information talks with clinics or rehabilitation facilities and support during these appointments, if desired •Assistance in applications (e.g., for employment participation benefits) •Online coaching to bridge the waiting period until treatment
2a	Vocational reintegration	•Workplace analysis •Analysis of personal strengths and weaknesses •Implementation of progressive reintegration
2b	Vocational reorientation	•Analysis of personal strengths and weaknesses •Lateral thinking in the professional context •Prepare or update application documents •Acquisition of internships, vocational training and development and jobs •Preparation for job interviews
3	Support	•Supporting the vocational reintegration •Exchange with health professionals •Connection to further social service providers •Contact person for questions concerning social and labor law •Support during appointments with the employer

Step 1: To work on one's own vocational reintegration requires a certain level of mental stability. Basis for that is reception of adequate mental health care. Therefore, the quality of a subjects' care was evaluated. If necessary, the RS case manager supported to implement an adequate health care by, for example, researching information about purveyors in the vicinity or by contacting health care professionals on the subjects' behalf and making appointments or by helping with bureaucratic matters. If needed, the RS psychologist bridged the waiting period until treatment with coaching. The following steps varied dependent on subjects' mental stability and personal priorities.

Step 2a: If a place of work was still available, intervention was oriented toward reintegration at the same employer. A workplace analysis was made to uncover obstacles for return to work which were then discussed with both the employer and the subject. If possible, reintegration was implemented in cooperation with the occupational reintegration management.

Step 2b: If no place of work was available, lateral thinking was used to create new occupational perspectives with the subject. The RS case manager helped to find possible new jobs in line with the specific talents of the subject, supported to prepare or update application documents and offered job interview trainings.

Step 3: The vocational reintegration or the start of a new job, if applicable, were accompanied by the RS until the end of the intervention. The RS case manager served as contact person for personal concerns and for matters concerning social and labor law. If needed, the RS psychologist supported with online coaching and mediation.

### Variables

Methods used by the RS were tallied and categorized.

Psychometrics of the subjects were measured using the following instruments:

- **Beck Depression Inventory** (BDI-II) (19).
- **Brief Symptom Inventory** (BSI) (20): The Global Severity Index (BSI-GSI) was used for further analysis. Additionally, the subscales Somatization (BSI-Soma), Depression (BSI-Depr) and Anxiety (BSI-Anx) were examined.
- **World Health Organization Quality of Life** (WHOQOL) (21). Psychological quality of life (QOL-psych) was measured using only the corresponding domain scores. General quality of life (QOL-general) was assessed with the first two questionnaire items: “How would you rate your quality of life? How satisfied are you with your health?”
- Work ability was measured using the German version (22) of the **Work Ability Index** [WAI; (23)]. The WAI is the most established measure of work ability (24) and also abbreviated versions have been shown to be reliable and highly correlated to the overall scale score (25). The selected item operationalized subjects' subjective work ability with the following question: “Assume that your work ability at its best has a value of 10 points. How many points would you give your current work ability?” Response format was a 10-point Likert scale ranging from 0 = *completely unable to work* to 10 = *very good work ability*.
- As an indicator for **return to work** (RTW) the following WAI item was examined: “How many whole days have you been off work because of a health problem (disease or health care or for examination) during the past year (12 months)?” Responses format was a five-point Likert scale with 1 = *none at all*, 2 = *at the most 9 days*, 3 = *10–24 days*, 4 = *25–99 days* and 5 = *100–365 days*.

### Statistical Analyses

Statistical advisory and verification of statistical procedures were conducted by the Institute for Biometrics of the Hannover Medical School. Data was analyzed using SPSS® 25 (IBM Corporation, Armonk NY, USA) for Windows®. Single missing scale values were considered to be missing at random and dealt with according to the respective handbook's guidelines. The experimental variables were evaluated according to the scales handbooks.

To assess changes over time and between and within the intervention and control group a series of ANOVAs for repeated measures was performed with the factors time (T0, T1, and T2) and group (intervention and control). Bonferroni adjustment for multiple comparisons was employed. The assumption of sphericity was not met for few ANOVAs and, in these cases, the Greenhouse Geisser statistics were reported. Effect sizes (ES) for the ANOVAs were reported as partial eta squared (η_p_^2^) with η_p_^2^ < 0.059 indicating small (S), 0.06 < η_p_^2^ < 0.14 indicating medium (M), and η_p_^2^ > 0.141 indicating large (L) effect sizes. The effect sizes within groups and across measurement points were reported as Cohen's *d* with 0 < *d* <0.19 indicating trivial (T), 0.20 < *d* <0.49 indicating small (S), 0.50 < *d* <0.79 indicating medium (M), and *d* > 0.80 indicating large (L) effect sizes.

### Ethics Committee Approval

The study was approved by the Ethics Committee of the Hannover Medical School (approval number: 3679-2017).

## Results

The sociodemographic information of the intervention and control group is displayed in Table 2. Table 3 lists the inference statistics. Table 4 shows the descriptive statistics of the experimental variables and Table 5 shows the mean comparison across measurement points within the experimental and control group.

**Table 2:** Sociodemographic information, separately for participants in the experimental and control group.

	**Groups**		
	**Exp**	**Control**	**Statistics**
*N*	41	41	
Age range in years	24–57	29–52	
Age range in years *M* (*SD*)	39 (8)	42 (7)	*t*_(77)_ = −1.72, *p* = 0.089
Gender (female/male)	29/10	25/15	*X*^2^(1) = 1.28, *p* = 0.257
Educational level (10 years/12+ years)	25/14	24/15	*X*^2^(1) = 0.05, *p* = 0.815
Civil status (single/partnership/divorced)	12/25/2	9/24/6	*X*^2^(2) = 2.45, *p* = 0.294
Sick days from onset of psychiatric problems to initial sick certificate (days)	1148	1013	*t*_(34)_ = 0.276, *p* = 0.758
Citizenship (German/other)	39/0	39/0	

*M, mean; SD, standard deviation. N does not necessarily add up to 41 due to missing values*.

**Table 3:** Inferential statistics of the experimental variables with the factor time (T0, T1, and T2).

	**Factor: Time**			
	**Exp**		**Control**	
	***F*(df)**	**η_**p**_^2^**	***F*(df)**	**η_**p**_^2^**
BDI-II	*F*_(2, 58)_ = 6.077^*^	0.173 [L]	*F*_(2, 72)_ = 3.275^*^	0.083 [M]
QOL-psych	*F*_(2, 56)_ = 8.016^*^^G^	0.223^G^ [L]	*F*_(2, 66)_ = 3.467^*^	0.095 [M]
QOL-general	*F*_(2, 58)_ = 6.187^*^	0.176 [L]	*F*_(2, 70)_ = 2.770^G^	0.073^G^ [M]
BSI-GSI	*F*_(2, 58)_ = 15.263^***^	0.345 [L]	*F*_(2, 72)_ = 6.315^*^	0.149 [L]
BSI-Soma	*F*_(2, 58)_ = 9.390^***^	0.245 [L]	*F*_(2, 70)_ = 3.102	0.081 [M]
BSI-Depr	*F*_(2, 58)_ = 12.997^***^	0.309 [L]	*F*_(2, 72)_ = 3.337^*^	0.085 [M]
BSI-Anx	*F*_(2, 58)_ = 9.600^***^	0.249 [L]	*F*_(2, 70)_ = 4.575^*^	0.116 [M]
WAI	*F*_(2, 54)_ = 9.703^***^	0.264 [L]	*F*_(2, 68)_ = 0.589	0.017 [S]
RTW	*F*_(2, 54)_ = 13.687^***^	0.336 [L]	*F*_(2, 60)_ = 1.615^G^	0.051^G^ [S]

*S, small effect size; M, medium effect size; L, large effect size*.

* *p <0.05,*

*** *p <0.001;*

G *Greenhouse-Geisser corrected statistics*.

**Table 4:** Descriptive statistics, separately for assessment time (T0, T1, and T2) and group (intervention vs. control).

	**T0**		**T1**		**T2**	
	**Exp *M* (*SD*), *N***	**Control *M* (*SD*), *N***	**Exp *M* (*SD*), *N***	**Control *M* (*SD*), *N***	**Exp *M* (*SD*), *N***	**Control *M* (*SD*), *N***
BDI-II	25 (12), 39	28 (15), 41	20 (11), 36	25 (15), 39	19 (13), 30	27 (16), 37
QOL-psych	39 (16), 37	36 (23), 41	46 (19), 34	40 (25), 38	51 (24), 30	44 (25), 35
QOL-general	28 (14), 39	32 (17), 41	35 (15), 36	33 (19), 39	40 (17), 30	39 (20), 36
BSI-GSI	1.5 (0.8), 39	1.8 (1.0), 41	1.1 (0.7), 36	1.4 (0.9), 39	1.1 (0.8), 30	1.6 (1.1), 37
BSI-Soma	1.2 (0.9), 39	1.4 (1.1), 40	0.9 (0.6), 36	1.1 (1.0), 39	0.8 (0.7), 30	1.4 (1.2), 37
BSI-Depr	1.7 (1.0), 39	2.0 (1.2), 41	1.2 (0.8), 36	1.7 (1.3), 39	1.2 (1.0), 30	1.9 (1.3), 37
BSI-Anx	1.5 (1.0), 39	1.6 (1.1), 41	1.1 (1.0), 36	1.2 (1.1), 39	1.0 (0.9), 30	1.5 (1.1), 36
WAI	2.4 (2.4), 37	3.9 (3.1), 41	4.0 (2.9), 36	3.8 (3.2), 39	5.0 (3.2), 29	4.2 (3.0), 35
RTW	5.0 (1.0), 38	3.6 (1.4), 37	3.6 (1.7), 32	3.3 (1.5), 35	3.4 (1.6), 29	3.2 (1.5), 34

*M, mean; SD, standard deviation*.

**Table 5:** Effect sizes for mean comparisons across measurement points within the experimental and control group.

	**Baseline to T1**		**Baseline to T2**		**T1 to T2**	
	**Exp Cohen's *d***	**Control Cohen's *d***	**Exp Cohen's *d***	**Control Cohen's *d***	**Exp Cohen's *d***	**Control Cohen's *d***
BDI-II	0.434 [S]	0.200 [S]	0.482 [S]	0.065 [T]	0.084 [T]	−0.129 [T]
QOL–psych	−0.400 [S]	−0.167 [T]	−0.601 [M]	−0.334 [S]	−0.233 [S]	−0.160 [T]
QOL-general	−0.483 [S]	−0.056 [T]	−0.781 [M]	−0.379 [S]	−0.314 [S]	−0.308 [S]
BSI-GSI	0.531 [M]	0.420 [S]	0.500 [M]	0.191 [T]	0.000 [T]	−0.200 [S]
BSI-Soma	0.389 [S]	0.285 [S]	0.488 [S]	0.000 [T]	0.155 [T]	−0.272 [S]
BSI-Depr	0.454 [S]	0.240 [S]	0.500 [M]	0.080 [T]	0.000 [T]	−0.154 [T]
BSI-Anx	0.400 [S]	0.364 [S]	0.522 [M]	0.091 [T]	0.105 [T]	−0.273 [S]
WAI	−0.602 [M]	0.032 [T]	−0.936 [L]	−0.098 [T]	−0.329 [S]	−0.129 [T]
RTW	1.026 [L]	0.207 [S]	1.237 [L]	0.276 [S]	0.121 [T]	0.067 [T]

*S, small effect size; M, medium effect size; L, large effect size*.

### Sample Characteristics

The distributions of age and marital and education status approximately correspond to the distribution of compulsorily insured employees in Germany (26). In both groups there was an unequal gender distribution toward a female majority which is comparable to the German and EU wide trend that women have a higher incidence to suffer from depression and anxiety than men (27). In both groups all of the subjects indicated to own the German citizenship, except for the missing values. All cases had a similar amount of sick days from the subjective onset of psychiatric problems up to initial sick note. There were no significant differences between the two groups on all above-mentioned variables (α ≤ 0.05).

The allocated 80 h of the intervention were roughly divided equally into occupational and psychological methods. With 43% a large part of the intervention concerned psychological matters such as stress management, social competence and job application trainings, psychoeducation, coaching and problem analyses. The case manager spent approximately 5% on initial assessment, 4% on the implementation of an adequate health care network, 12% on home visits and 5% on career services. Seventeen percent were spent on rehabilitation management. The remaining hours of the intervention were spent on conversations with the subjects, clinicians, social service providers and others (7%) bureaucratic matters, preparation and follow-up (8%).

### Depressive Symptoms: BDI-II

**Figure 2:**
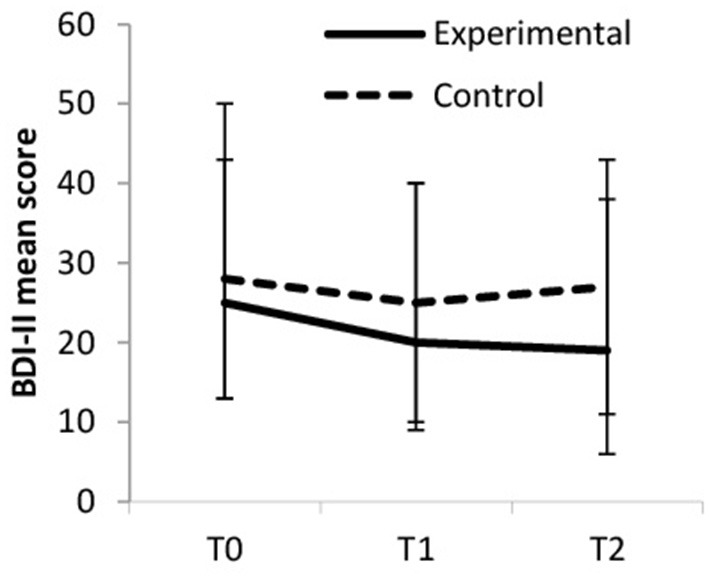
Beck depression inventory (BDI-II).

BDI-II mean scores decreased over time, but more so in the experimental group (large ES) compared to the control group (medium ES). Within the experimental group, the symptoms of depression decreased from baseline to T1 (small ES) and from baseline to T2 (small ES); from T1 to T2, the symptoms of depression remained stably low (trivial ES). Within the control group, depressive symptoms decreased from baseline to T1 (small ES) but increased again from T1 to T2 (trivial ES); from T1 to T2, the symptoms of depression remained about the same (trivial ES). Between-group differences were significant at T2 [*F*_(1, 65)_ = 5.166, *p* = 0.026, η_p_^2^ = 0.074]. Figure 2 displays the changes in BDI-II scores.

### Psychological Quality of Life

**Figure 3:**
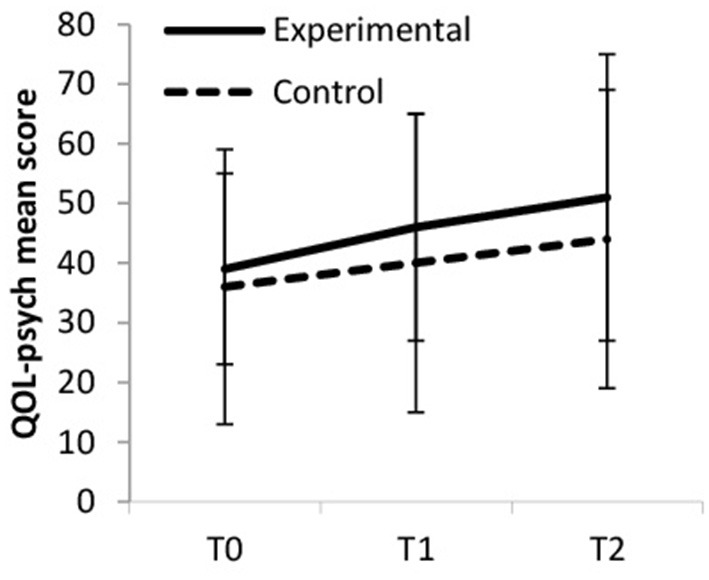
Psychological quality of life (QOL-psych).

Psychological QOL increased over time, but more so in the experimental group (large ES) compared to the control group (medium ES, see Figure 3). Within the experimental group, psychological quality of life increased from baseline to T1 (small ES) from baseline to T2 (medium ES) and from T1 to T2 (small ES). Within the control group psychological quality of life also increased but to a lesser extend from baseline to T1 (trivial ES), from baseline to T2 (small ES), and from T1 to T2 (trivial ES). Between-group differences were significant at T2 [*F*_(1, 65)_ = 6.178, *p* = 0.016, η_p_^2^ = 0.087].

### General Quality of Life

**Figure 4:**
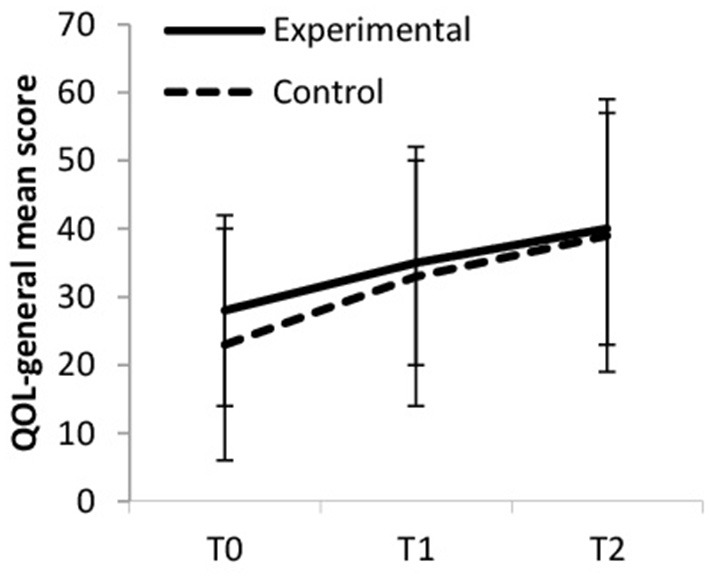
General quality of life (QOL-general).

General QOL increased over time, but more so in the experimental group (large ES) compared to the control group (medium ES, see Figure 4). Within the experimental group, general quality of life increased from baseline to T1 (small ES), from baseline to T2 (medium ES) and from T1 to T2 (small ES). Within the control group general quality of life stayed rather constant from baseline to T1 (trivial ES), and increased from baseline to T2 and from T1 to T2 (small ES). Between-group differences were significant at T2 [*F*_(1, 65)_ = 5.166, *p* = 0.026, η_p_^2^ = 0.074).

### Psychological Strain

**Figure 5:**
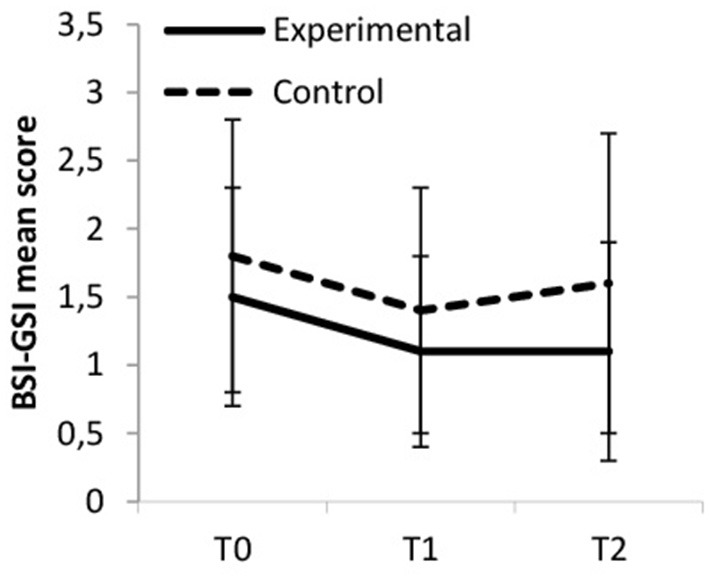
Overall psychological strain (BSI-GSI).

Overall psychological strain, as measured by the BSI-GSI, decreased over time, and more so in the experimental group compared to the control group, though both effect sizes were classified as large. Within the experimental group, BSI-GSI scores decreased from baseline to T1 and from baseline to T2 (medium ES); from T1 to T2 they remained fairly constant (trivial ES). Within the control group general psychological strain decreased from baseline to T1 but increased again from T1 to T2 (small ES). Hence, the effect from T0 to T2 was trivial. Between-group differences were significant at T2 [*F*_(1, 65)_ = 6.178, *p* = 0.016, η_p_^2^ = 0.087]. Figure 5 displays the changes in BSI-GSI scores.

Regarding the BSI subscales the pattern of changes of the experimental and control group across measurement points were comparable between the subscales for Somatization, Depression and Anxiety: Values decreased, but more so for the experimental group (large ES) compared to the control group (medium ES). Within the experimental group scores decreased from baseline to T1 (small ES) and from baseline to T2 (medium ES for BSI-Depr and BSI-Anx and nearly medium ES for BSI-Soma); scores remained stably low from T1 to T2 (trivial ES). Within the control group scores decreased from baseline to T1 (small ES) but increased again from T1 to T2 (small ES for BSI-Anx and BSI-Soma and trivial ES for BSI-Depr). Hence, the effect sizes from T0 to T2 were trivial. Between-group differences were significant for BSI-Soma at T2 [*F*_(1, 64)_ = 5.791, *p* = 0.019, η_p_^2^ = 0.083] and for BSI-Depr at T1 and T2 [*F*_(1, 65)_ = 4.355, *p* = 0.041, η_p_^2^ = 0.063 and *F*_(1, 65)_ = 5.471, *p* = 0.022, η_p_^2^ = 0.078, respectively].

### Work Ability

**Figure 6:**
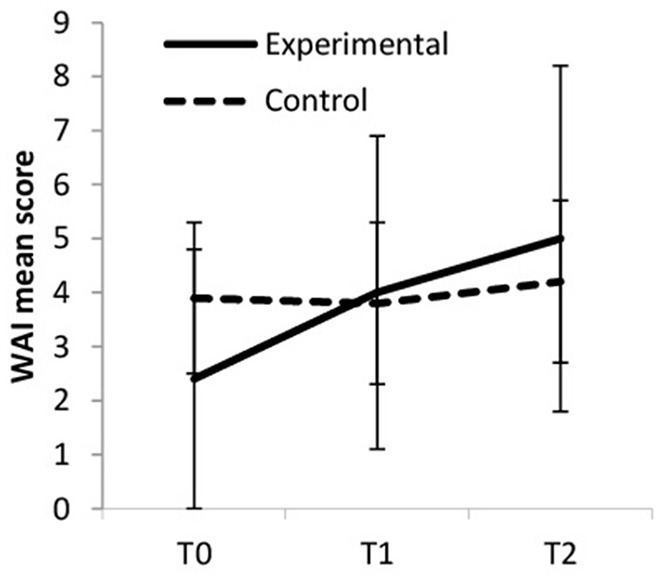
Work ability index (WAI).

Work ability increased over time, but more so in the experimental group (large ES) compared to the control group (small ES, see Figure 6). Within the experimental group, work ability increased from baseline to T1 (medium ES), from baseline to T2 (large ES), and from T1 to T2 (small ES). Within the control group work ability remained fairly stable from baseline to T1 (trivial ES) and increased slightly from T1 to T2 (trivial ES). The difference between baseline and T2 was also trivial. Between-group differences were significant at T0 [*F*_(1, 61)_ = 4.454, *p* = 0.039, η_p_^2^ = 0.068].

### Return to Work

RTW was measured by the number of days absent from work throughout the past year. Absentee days decreased in both groups, but more so in the experimental group (large ES) compared to the control group (small ES). In the experimental group, absenteeism decreased from baseline to T1 (large ES) and from baseline to T2 (large ES); from T1 to T2 it remained rather constant (trivial ES). In the control group absenteeism stayed rather unchanged from T0 to T1 (small ES), from T0 to T2 (small ES) as well as from T1 to T2 (trivial ES). Between-group differences were significant at T0 [*F*_(1, 57)_ = 31.212, *p* = 0.000, η_p_^2^ = 0.354].

## Discussion

Even though various interventions exist for patients in danger of permanent disability due to mental illness, almost no target those in which this risk has materialized. In the present study this specific group was addressed and received a novel intervention. The intervention was tailored towards individual needs, included close monitoring and connected the various practitioners involved in the case with the goal of mental health improvement and vocational reintegration.

Experimental subjects showed improved mental health after 12 months while subjects of the control group did not. More specifically, depressive symptoms declined continuously in the experimental group and so did general psychological strain: Somatization, anxiety and depression all consistently receded in the experimental group, but did not so among controls. Psychological and general quality of life increased in both groups over time, but more so in the experimental group compared to the control group. Work ability improved much more robustly among experimental subjects. Likewise, return to work improved in the experimental group as days missed at work due to mental illness decreased.

Clearly, the intervention had a positive effect on all mentioned variables; effect sizes for the factor time were consistently large and significant in the experimental group. The focus on the amelioration of the mental constitution as well as work-related factors seems to be superior to no intervention and waiting time alone. Having a contact person for personal concerns that helps with the management of treatments and work related issues seems to be highly beneficial even for those in premature retirement.

Particularly, work ability increased considerably. With the holistic treatment administered in this intervention and improved mental health higher levels of work ability can be reached and the resumption of work is more likely. In this study return to work was optional and not explicitly addressed or measured. Nonetheless, days missed at work decreased within the observation period in the experimental group.

### Limitations

Despite these promising results some remarks have to be made concerning sample characteristics and study design. Strictly speaking these results can only be applied in societies that have some form of statutory pension insurance and occupational disability insurance as the contact between the subject and their insurer was the starting point of this intervention. Also, the results can only be applied to those who realize that their mental illness may be the cause for their problem.

While the main goal, return-to-work, was fully standardized, the individual steps to reach it were not. Instead, it consisted of 80 h of all that psychiatry had to offer. The authors chose this approach because, after all, at the time of recruitment all subjects had had standardized interventions that had failed. Full standardization couldn't have yielded any information about the individual obstacles of therapeutic success. The single steps of the intervention are, nonetheless, comprehensive and transparent.

The assumption of equal variances did not hold for few ANOVA calculations. Outcomes were reported nonetheless and corrected values were used in these cases.

At first sight the method of recruitment may provoke the question if the control group and the experimental group are comparable as the subjects in each group had contacted their insurer at a different time. While subjects in the control group had subjectively been suffering from mental illness for an average of 10.7 years before being included in the study, subjects in the experimental group had this for an average of 5.5 years. However, there is no logical explanation of increased chronicity in the control group as chronicity can already be expected after an even shorter period. Alienation from the workplace and social disintegration happen much quicker than either 5 or 10 years. Both groups also had equally bad functioning at the start of the study. Therefore, the aspect of how long the occupational disability had lasted at the start of the study might not be primarily relevant. Maybe the subjects in the control group felt more hopeless than the subjects in the experimental group, which may have caused the effect of the intervention to be over-estimated. This, of course, could not be controlled for as giving hope was one of the aims of the intervention. The problem of the different group characteristics also couldn't have been approached with a different sampling strategy, because it wasn't available for ethical reasons.

All in all, it could be shown that the intervention had a positive effect on quality of life, psychological strain, depressive symptoms, work ability and return to work over the course of the 12-months period. Individual support and coaching with the goal to relief psychological strain and promote occupational reintegration was shown to be beneficial even for those who went through all previous interventions unsuccessfully. In the long run, these subjects will likely need less health services and pension payments, which makes the intervention the preferable option from a total cost perspective.

The question arises whether the positive effects of the intervention last throughout the years. A follow-up study is currently being conducted in which cases are observed 1 year after termination of the intervention. First results are promising that the positive effects of the intervention persist.

Future research should examine additional variables that might influence the effect of post-retirement interventions on mental health improvement and vocational reintegration. For example, monetary variables should be investigated. The precise interaction between monetary incentive of pensioning and reactivation is still unclear. Previous results suggest that financial security during a period of mental illness is indeed helping rehabilitation, while poverty is associated with a worse outcome (2). Moreover, the quality of the interventions that subjects had received before the time of observation was not evaluated. Psychiatric help is not available everywhere and always, but this is a problem for a separate study. Furthermore, a more concise measure of return to work could be provided.

## Conclusion

The results of the present study show that there is still potential for those who are ejected from the workforce and social context after premature retirement. Individualized and holistic treatment, as administered in this study, can improve psychological well-being and work ability. Eventual stabile occupational reintegration and placement into a problem-compatible value-generating new job reverses the money flow, which is beneficial for the individual and society. But even if full occupational recovery cannot be reached, the mere improvement of persons' living situation is an outcome worth striving for. The authors have the opinion that psychological well-being is fundamental for sustainable recovery, even if it may not cause a return to work.

## Data Availability Statement

The raw data supporting the conclusions of this article will be made available by the authors, without undue reservation.

## Ethics Statement

The studies involving human participants were reviewed and approved by Ethics Committee of the Hannover Medical School. The patients/participants provided their written informed consent to participate in this study.

## Author Contributions

EB-W collected and analyzed the data and wrote the manuscript. FW designed the study, interpreted the data, and wrote the manuscript. Both authors have approved the final version of the manuscript.

## Conflict of Interest

The authors declare that the research was conducted in the absence of any commercial or financial relationships that could be construed as a potential conflict of interest.
